# Renal abscess in a patient with a long-term double-J stent due to *Candida albicans*

**DOI:** 10.1590/0037-8682-0197-2024

**Published:** 2024-08-09

**Authors:** Kemal Buğra Memiş, Muktedir Emir Şahin, Volkan Kızılgöz, Ali Osman Gülmez, Sonay Aydın

**Affiliations:** 1 Erzincan University, School of Medicine, Department of Radiology, Erzincan, Turkey.

**FIGURE 1:**
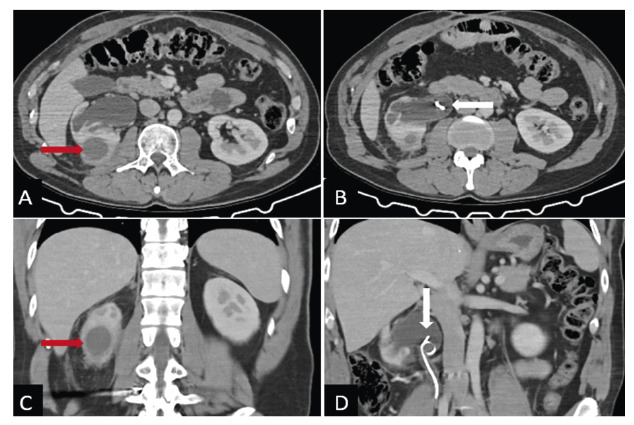
Axial **(A-B)** and coronal **(C-D)** plane enhanced abdomen computed tomography shows a well-defined collection resembling an abscess in the right kidney. The collection has a diameter of 3 cm at its lower pole **(red arrows)**. Additionally, grade 3 hydronephrosis and double-J stent are observed in the right kidney **(white arrows)**.

**FIGURE 2:**
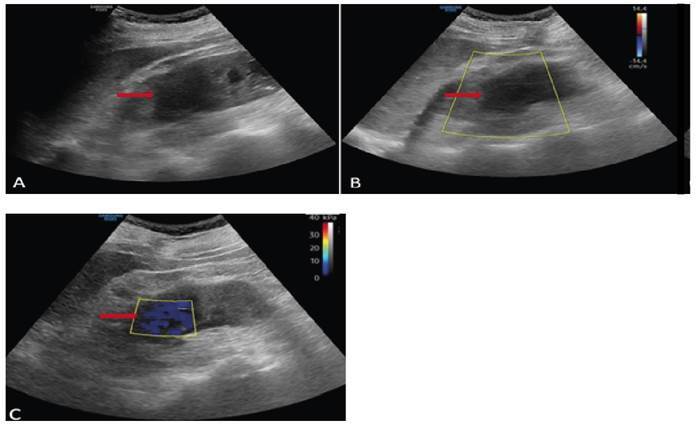
Longitudinal gray scale **(A),** color Doppler **(B)** ultrasound, and shear-wave elastography **(C)** images of the lesion in the right kidney. Doppler ultrasonography shows a hypoperfused lesion in the renal parenchyma of the right kidney. The sonoelastography images showed the soft stromal structures **(red arrows)**.

A 39-year-old male patient underwent right hemicolectomy and double-J stent placement for penetrating abdominal trauma that resulted in ureter and bowel injuries. Contrast-enhanced abdominal computed tomography revealed grade 3 hydronephrosis of the right kidney and a double-J catheter. Furthermore, a substantial amount of intrarenal fluid was detected in the lower pole of the right kidney, leading to decreased perfusion of the surrounding renal tissue and inflammation of the pararenal adipose tissue (Figure 1). Sonoelastography of the right kidney showed that the lesion predominantly consisted of soft stromal structures (Figure 2). Treatment was initiated specifically upon *Candida albicans* detection in the urine culture. Repeated imaging revealed a reduction in the lesion size, and complete recovery was achieved without percutaneous drainage.

*Candida albicans* is a component of the normal microflora in the gastrointestinal tract of humans^1^. The disruption of the skin and digestive barriers can lead to invasive diseases^2^ ^,^ ^3^. In healthy adults, *Candida* is detected in less than 1% of clean urine samples; however, in hospitals, it is typically detected in 5%-10% of positive urine cultures, particularly in patients with bladder catheters^4^ ^,^ ^5^. *Candida*-related renal abscesses are characterized by localized pus in the renal parenchyma and often present with fever, flank pain, and septicemia^2^ ^,^ ^3^ ^,^ ^6^. Prompt imaging and antifungal treatments, such as fluconazole, are essential to prevent complications^6^. Candida infections should be considered in complex renal lesions in patients with a history of bladder catheterization or gastrointestinal perforation.
